# An Assessment of Climate Induced Increase in Soil Water Availability for Soil Bacterial Communities Exposed to Long-Term Differential Phosphorus Fertilization

**DOI:** 10.3389/fmicb.2020.00682

**Published:** 2020-05-15

**Authors:** Kate C. Randall, Fiona Brennan, Nicholas Clipson, Rachel E. Creamer, Bryan S. Griffiths, Sean Storey, Evelyn Doyle

**Affiliations:** ^1^School of Biology and Environmental Science, Earth Institute, University College Dublin, Dublin, Ireland; ^2^School of Life Sciences, University of Essex, Colchester, United Kingdom; ^3^Teagasc Environment Research Centre, Wexford, Ireland; ^4^Soil Biology Group, Wageningen University & Research, Wageningen, Netherlands; ^5^SRUC, Crop & Soil Systems Research Group, Edinburgh, United Kingdom

**Keywords:** climate change, soil moisture, phosphorus, bacteria, rhizosphere

## Abstract

The fate of future food productivity depends primarily upon the health of soil used for cultivation. For Atlantic Europe, increased precipitation is predicted during both winter and summer months. Interactions between climate change and the fertilization of land used for agriculture are therefore vital to understand. This is particularly relevant for inorganic phosphorus (P) fertilization, which already suffers from resource and sustainability issues. The soil microbiota are a key indicator of soil health and their functioning is critical to plant productivity, playing an important role in nutrient acquisition, particularly when plant available nutrients are limited. A multifactorial, mesocosm study was established to assess the effects of increased soil water availability and inorganic P fertilization, on spring wheat biomass, soil enzymatic activity (dehydrogenase and acid phosphomonoesterase) and soil bacterial community assemblages. Our results highlight the significance of the spring wheat rhizosphere in shaping soil bacterial community assemblages and specific taxa under a moderate soil water content (60%), which was diminished under a higher level of soil water availability (80%). In addition, an interaction between soil water availability and plant presence overrode a long-term bacterial sensitivity to inorganic P fertilization. Together this may have implications for developing sustainable P mobilization through the use of the soil microbiota in future. Spring wheat biomass grown under the higher soil water regime (80%) was reduced compared to the constant water regime (60%) and a reduction in yield could be exacerbated in the future when grown in cultivated soil that have been fertilized with inorganic P. The potential feedback mechanisms for this need now need exploration to understand how future management of crop productivity may be impacted.

## Introduction

Soil is an invaluable commodity urgently in need of protection if we are to support the growing global human population (Arneth et al., 2019). This is particularly poignant in the face of climate change, as 95% of our food comes directly or indirectly from soil (FAO, 2015; Spanner, 2015), with a 70% increase in demand predicted by 2050 (Dubey et al., 2019). Grassland cultivation has been a tool for increasing land used for agricultural purposes (Qi et al., 2012) and management of these systems has steadily intensified since the 1960’s (Isselstein et al., 2005). As this looks set to continue, optimizing future grassland management, whilst sustaining a healthy soil environment is therefore vital (Lemanceau et al., 2015).

A key component to sustaining soil health is through the soil microbiome (Glick, 2018; Dubey et al., 2019). As the drivers of biogeochemical nutrient cycling, microbes disproportionately influence soil processes, plants and wider ecosystem functioning (Falkowski et al., 2008; Jansson and Hofmockel, 2019), and can form a range of symbioses (Lambers et al., 2009). However, climate change impacts on microbial communities, and the repercussions this may cause for humanity are not well understood, despite recent efforts to promote awareness (Cavicchioli et al., 2019; Hartman and Tringe, 2019; Jansson and Hofmockel, 2019; Timmis et al., 2019).

Across Atlantic Europe, robust increases in heavy precipitation are predicted for both winter and summer months (IPCC, 2014). With increased rainfall and flooding, land degradation (Arneth et al., 2019) and soil erosion can be exaggerated, particularly for agricultural soils where management has weakened structure (St Clair and Lynch, 2010). Excessive periods of waterlogging can increase nutrient loss via overland flow (Di and Cameron, 2002; McDowell et al., 2003), impact root development and soil nutrient availability (St Clair and Lynch, 2010; Hamonts et al., 2013). As a result, fertilization of soil and the feedback onto plant productivity will likely be effected by heightened periods of rainfall. For inorganic phosphorus (P) fertilization, this is particularly concerning due to the political, environmental and economic issues already dominating P resource availability and production (Elser et al., 2007; EU Commission, 2011; Ceci et al., 2018).

As concerns surrounding inorganic P availability within soil have increased, research exploring the soil microbiota as a sustainable tool for P mobilization has also increased. Whilst some studies report an insensitivity of soil microbiota to conservative application rates of inorganic P (Beauregard et al., 2010; Wakelin et al., 2012; Gosling et al., 2013; Chen et al., 2014), bacterial communities appear particularly responsive when soils are unfertilized (Mander et al., 2012; Tan et al., 2013; Randall et al., 2019). This has revealed bacterial taxa with potential roles in improving soil P mobilization (Mander et al., 2012; Lakshmanan et al., 2014; Randall et al., 2019), however, study of microbial responses to elevated soil moisture content and P fertilization within temperate regions is unexplored.

To-date, soil microbial studies concerning increased soil moisture focus largely on the effect of re-wetting arid soils and the microbiome of flooded paddy fields (Fierer et al., 2003; Austin et al., 2004; Huxman et al., 2004; Liu et al., 2009). In such scenarios, microbial community structure and activity profiles have been found to significantly differ between flooded and unflooded soils (Kimura and Asakawa, 2006; Breidenbach and Conrad, 2014), with reductions in microbial biomass, aerobic bacterial and mycorrhizal fungal PLFA biomarkers also being observed (Unger et al., 2009). In addition, microbial functional responses have shown an increased abundance of denitrifiers in soil exposed to elevated water availability (Liu et al., 2014), as well as increased production of the greenhouse gases (GHG) CH_4_ and N_2_O, to which microbes contribute significantly (Ferré et al., 2012; Tete et al., 2015). With more frequent and heavier precipitation events, conditions imposed upon temperate regions may select for soil anaerobic bacteria and archaea, the likes which dominate paddy fields post flooding (Liesack et al., 2000; Reim et al., 2012). Such consequences may impact future GHG emissions, as demonstrated when temperate coastal grasslands flood (Gebremichael et al., 2017).

The aim of this study was to test the hypothesis that a long-term difference in inorganic P fertilization, combined with a short-term difference in soil water availability and plant presence will significantly affect soil bacterial community composition, plant biomass and soil enzymatic activity (acid phosphomonoesterase and dehydrogenase activity). To achieve this, a mesocosm, growth chamber study was conducted.

## Materials and Methods

### Soil Sampling

Soil was sampled from a long established (44 years), inorganic P grazed field trial located in Co. Wexford, Ireland (52°16′ N, 06°30′ W, during August 2020). Details are documented by Randall et al. (2019). Soil from three continually unfertilized (0 kg P ha^–1^ y^–1^) (P0) and three continually fertilized field plots, (30 kg P ha^–1^ y^–1^) (P30) was sampled (Supplementary Figure 1), ensuring consistent soil texture (Tunney et al., 2010). For each field plot, 25 soil cores were sampled from the top 20 cm of the soil profile using a Dutch auger (4 cm diameter) during August 2012. A composite sample was then made for each field plot by sieving (2 mm) the 25 cores and homogenizing, producing six composite soil samples. Baseline biochemical and physico-chemical properties were then determined. The remainder of each composite sample was stored in separate containers and left to settle in the dark for 1 week at 15°C (Zeng, 2017; Comeau et al., 2018; Randle-Boggis et al., 2018).

### Experimental Set-Up

**FIGURE 1:**
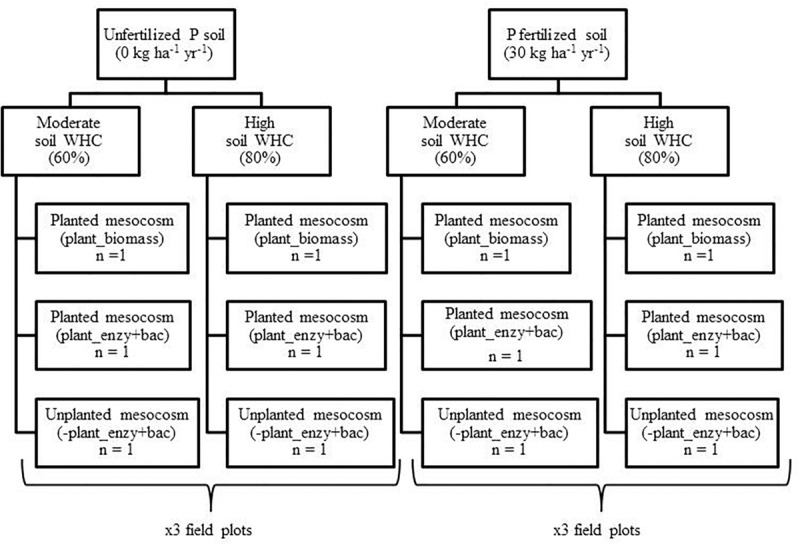
Design of the mesocosm growth chamber experiment. Field soil was sampled from a long-term inorganic phosphorus (P) field trial (44 years), from unfertilized (P0) (*n* = 3) and fertilized (P30) (30 kg ha^–1^ y^–1^) (*n* = 3) field plots. Soil water holding capacity (WHC) was then manipulated to either 60% or 80% and was maintained. Mesocosms were either sown with an individual seed of spring wheat *(T. aestivum)*, Trappe variety (Goldcrop Ltd., Co. Cork, Ireland), or remained unplanted, serving as negative plant controls. The experiment ran for four months within a growth chamber.

Mesocosms were constructed from plastic trunking (100 cm × 3.6 cm × 3.6 cm) (Radionics Ltd., Dublin). After 1 week of incubating the soil in the dark, the soil water holding capacity (WHC) of each composite field sample (3 × P0 and 3 × P30) was determined (see Supplementary Material). The overall experimental design is presented in Figure 1.

For each of the six composite samples (3 × P0 and 3 × P30), the following protocol was performed; soil was packed into six pre-weighed mesocosms at a bulk density of 1.1 g cm^–3^ (Dijkstra and Cheng, 2007). For ease, the addition of soil was made in 10 cm sub-sections D1 (0–10 cm) to D8 (70–80 cm) (see Supplementary Material).

Within these six mesocosms, three then had the soil WHC adjusted to 60%, to reflect a moderate soil WHC (60%). The remaining three mesocosms had the soil WHC adjusted to 80%, representing a high soil water treatment (80%) (Chen et al., 2007; Dijkstra and Cheng, 2007; Wang et al., 2016). The final weight of each mesocosm was recorded and was maintained for the duration of the experiment using distilled water (Dijkstra and Cheng, 2007; Lazcano et al., 2014) (see Supplementary Material). The mesocosms detachable front cover provided access for watering and sampling (Supplementary Figure 2).

A single seed of spring wheat (*T. aestivum*) was then sown into two mesocosms, 5 cm below the surface. Two planted mesocosms were required so one could be used to determine total root biomass (+Plant_biomass), whilst the other was used to determine bulk soil enzymatic activity and bacterial community composition (+Plant_enzy + bac) (Figure 1). The third mesocosm remained unplanted to serve as a negative plant control. This set-up was repeated for the remaining five composite field samples, with the final design consisting of 36 mesocosms.

#### Growth Chamber Conditions

The experiment ran for a 4 month period and was conducted in a controlled growth chamber (Series 3; Temperature Applied Sciences Ltd.). Chamber parameters were set at 75% humidity with a diurnal light intensity program (16 h daylight, 8 h night) (Haworth et al., 2011, 2013). The temperature regime reflects the 1.5°C increase the IPCC wish to cap global warming (IPCC, 2018). A 1.5°C increase was added to the previous year’s (2011) mean monthly temperatures (March–June) recorded for the area (Met Éireann, 2011). The block temperatures used to simulate the 4 month growing season were, 8.3, 10.0, 12.5, and 16°C.

### Experimental Sampling Protocol

After 4 months, all 36 mesocosms were destructively sampled. At this stage, the plants had reached the end of their vegetative growth stage (Zadoks et al., 1974). All sampling was conducted under sterile conditions, with the front of the trunking removed to access soil within each mesocosm. All planted mesocosms were carefully inspected to identify depth of root growth within each of the 10 cm sub-sections (D1–D8). Across all planted mesocosms, roots were present up to sub-section D5.

#### Plant Biomass (+plant_biomass)

To determine the shoot and root biomass of spring wheat, one planted mesocosm per treatment, per original field plot was sampled (Figure 1, +plant_biomass). The intact plant was carefully removed from the soil. Shoots and roots were severed and the fresh weights of both were recorded. Roots and shoots were then individually oven dried for 48 h at 60°C to determine the dry weight.

#### Soil Bacterial Communities and Bulk Soil Enzymatic Activity (+plant_enzy + bac, –plant_enzy + bac)

The remaining planted and all unplanted mesocosms were sampled to determine bacterial community composition and bulk soil enzymatic activity (Figure 1, +plant_enzy + bac and –plant_enzy + bac).

For planted mesocosms, sampling was conducted separately for each sub-section where roots were present (D1–D5). Planted bulk and rhizosphere soil was also sampled within each sub-section for molecular characterization of bacterial communities. Rhizosphere soil was considered as soil adhering to root section post 1 min of shaking by hand. The remaining soil within the mesocosm was considered as the bulk soil. In unplanted mesocosms, the equivalent sub-sections (D1–D5) were sampled, with all soil considered as bulk. Soil samples intended for molecular analysis were then frozen (−20°C).

At the same time, additional bulk soil samples were collected within planted (+plant_enzy + bac) and unplanted (–plant_enzy + bac) mesocosm sub-sections (D1–D5) to determine both dehydrogenase and acid phosphomonoesterase activity and soil physico-chemcial properties.

### Soil Biochemical and Physico-Chemical Properties

For details of how to determine the gravimetric soil water content and WHC please refer to the Supplementary Material.

#### Soil pH

Soil pH was determined using a soil: deionized water ratio of 1:2 (w/v) (McCormack, 2002), using 5 g of fresh sieved soil and measured using a WTW pH526 pH meter (Labsource, United States).

#### Soil Available Inorganic Phosphorus

Soil available inorganic P was determined using the Morgan’s extraction method (McCormack, 2002) as described by Massey et al. (2012) and measured spectrophotometrically at 880 nm using the phosphomolybdate method (Murphy and Riley, 1962).

#### Dehydrogenase Activity

1 g of the freshly sampled soil was used to determine dehydrogenase activity, using the method described by Alef and Nannipieri (1995). The optical density of the supernatant was measured at 546 nm using a spectrophotometer (Helios Unicam, United Kingdom). Dehydrogenase activity was calculated using standard curves constructed with known concentrations of TPF and expressed as μg TPF g^–1^ dry soil 24 h^–1^.

#### Acid Phosphomonoesterase Activity

1 g of the freshly sampled soil was used to determine acid phosphomonoesterase activity using a modified method of Tabatabai and Bremner (1969), with the toluene step removed. The optical density of the supernatant was measured at 400 nm using a spectrophotometer (Helios Unicam, United Kingdom). Acid phosphomonoesterase activity was calculated with respect to standard curves from known concentrations of p-nitrophenol and expressed as μg p-NP g^–1^ soil h^–1^.

### DNA Extraction and 16S rRNA Bacterial Amplicon Library Creation

DNA extractions followed a modified version of the method by Griffiths et al. (2000) (Storey et al., 2014). Triplicate extractions were performed for each biological sample and pooled. DNA was extracted from the D1 section and the deepest section containing roots (D5). For planted mesocosms (+plant_enzy + bac), DNA was extracted from bulk and rhizosphere soil from both the D1 and D5 sub-sections. For the unplanted mesocosms (–plant_enzy + bac), DNA was extracted from soil within D1 and D5 sub-sections. Library preparation for high throughput sequencing (HTS) followed Randall et al. (2019).

### Bioinformatic Pipeline for High Throughput Sequencing (HTS) Data

After downloading the raw sequencing reads, the forward and reverse reads were combined into contigs using Mothur (Schloss et al., 2009) and uploaded to the NCBI SRA (PRJNA610548). Read processing and quality control (QC) followed Randall et al. (2019). After QC, identical sequences were grouped into “unique” sequences. Chimeric sequences were identified using the UCHIME algorithm within Mothur with 142,556 chimeric sequences removed. From 3,467,214 contigs, 1,723,604 paired end reads were constructed and 516,752 unique sequences detected. 201,430 sequences were assigned to operational taxonomic units (OTUs) using the “cluster” command and the average neighbor algorithm. OTU-based analyses were performed using a cutoff of 0.03. Taxonomy was assigned to the aligned sequences by comparing data to the SILVA database for bacteria (arb-silva.de/silva-license-information) and relative abundances were calculated.

### Statistical Data Analysis

All univariate analyses were performed using Minitab v15 (Minitab Ltd.). For the analysis of baseline soil biochemical and physico-chemical parameters (pH, available inorganic P, dehydrogenase and acid phosphomonoesterase activity) the design was an independent between-subjects design, with inorganic P fertilization rate (P0 vs. P30) as the independent variable.

Univariate data from the experiment was analyzed by applying general linear models (GLM) (Minitab Ltd.) to test main and interactive effects of the independent variables, inorganic P fertilization rate (P0 vs. P30), soil water content (60% vs. 80% soil WHC) and where appropriate, plant (unplanted vs. planted) on the dependent variables; spring wheat shoot and root biomass and bulk soil dehydrogenase and acid phosphomonoesterase activity. Significance levels were set at the 0.05 probability level.

Multivariate analyses to test for differences in beta diversity of bacterial assemblages were performed using PRIMER-E v6.1.10 with the PERMANOVA +add-on package (PRIMER-E Ltd., Plymouth, United Kingdom) (Anderson, 2001). The relative abundance data for bacterial communities were initially transformed to the 4th root (Clarke and Warwick, 2001). The dataset was sub-divided and analyzed to answer specific questions for paired samples (i.e., D1 vs. D5 sub-sections and bulk vs. rhizosphere soil) and Bray-Curtis resemblance matrices were generated (Clarke et al., 2006). Data was tested for homogeneity of variances at the lowest level of factor combinations using the PermDisp function in PRIMER-E and permutation analysis of variance (PERMANOVA) were conducted, applying 9,999 permutations of residuals under a reduced model.

## Results

### Baseline Soil Characteristics

Prior to the experimental manipulation, baseline field soil characteristics were determined (Table 1). A significant main effect of inorganic P fertilization rate was detected for soil pH, available inorganic P concentration and acid phosphomonoesterase activity (Table 1). Across all the plots, unfertilized (P0) soil had significantly lower pH [*t*_(__6__)_ = 23.09, *p* = 0.003] and available inorganic P [*t*_(__6__)_ = 123.00, *p* = 0.001] compared to fertilized (P30) soil (Table 1).

**TABLE 1:** Mean baseline soil pH, available inorganic phosphorus (P) concentrations and enzymatic activities measured in unfertilized (P0) (*n* = 3) and fertilized (P30) field soil (*n* = 3) from a long running (44 years) P fertilization trial (prior to experimental manipulation).

	**Inorganic P fertilization rate (kg P ha^–1^ y^–1^)**	
	**P0**	**P30**
pH	**5.33 (*0.01*)^a^**	**5.64 (*0.01*)^b^**
Available inorganic P (μg P g^–1^ dry soil)	**1.18 (*0.01*)^a^**	**7.22 (*0.07*)^b^**
Dehydrogenase activity (μg TPF g^–1^ dwt soil 24 h^–1^)	129.27 (*4.34*)^a^	126.41 (*14.59*)^a^
Acid phosphomonoesterase activity (μg PNP g^–1^ dwt soil h^–1^)	**17.22 (*0.31*)^a^**	**12.01 (*0.25*)^b^**

*Standard errors are shown in parentheses with significant differences between P treatment denoted by different letters (*p* < 0.05) and highlighted in bold.*

For baseline enzymatic activity measurements, whilst dehydrogenase activity did not significantly differ between unfertilized (P0) and fertilized (P30) soil (Table 1), acid phosphomonoesterase activity was significantly higher in the unfertilized (P0) soil compared to the fertilized (P30) soil [*t*_(__6__)_ = 5.00, *p* = 0.003] (Table 1).

### Aboveground and Belowground Spring Wheat Biomass at Harvest

**FIGURE 2:**
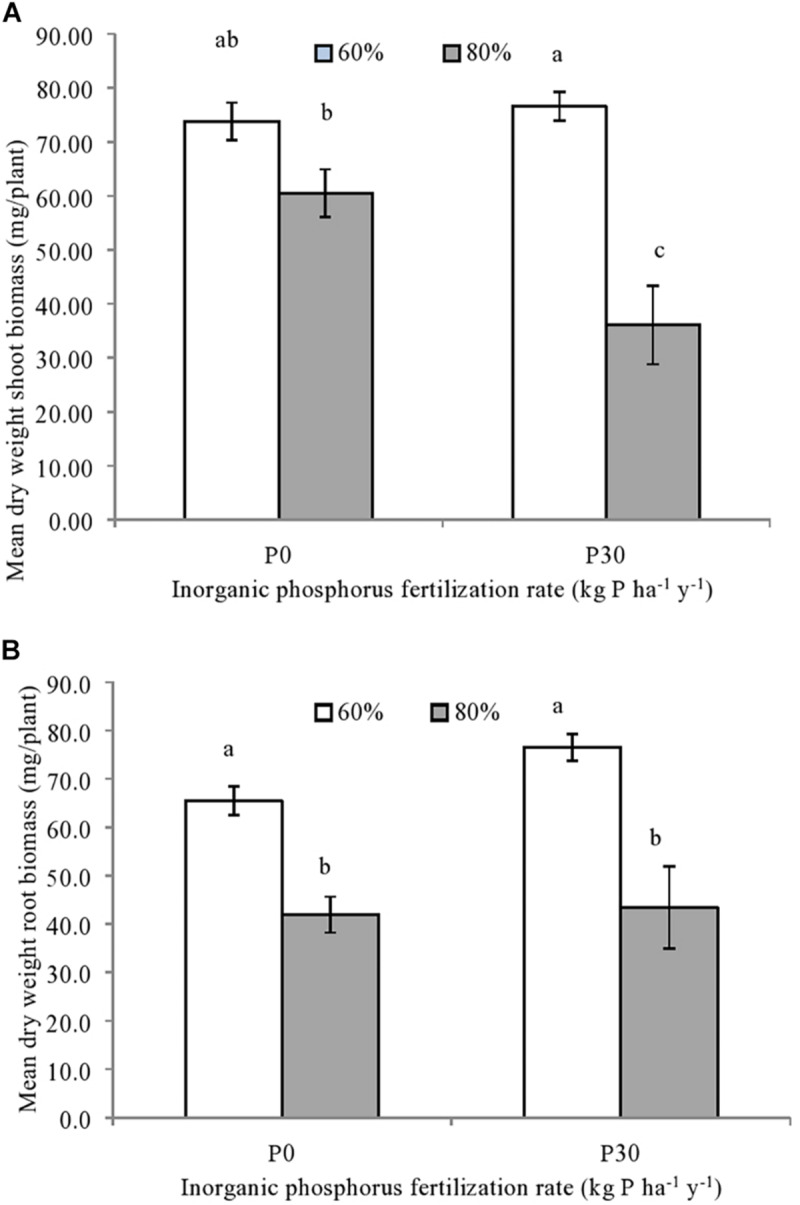
**(A)** Mean shoot and **(B)** root biomass of individually sown spring wheat *(T. aestivum)*, grown within mesocosms using field soil sampled from a long-term inorganic phosphorus (P) fertilization experiment (44 years). Soil used was sampled from field plots that were either unfertilized (P0) (*n* = 3) or fertilized at a rate of 30 kg P ha^–1^ y^–1^ (P30) (*n* = 3). Soil water holding capacity was altered to 60% or 80% and was maintained within a growth chamber for four months. Error bars represent standard error of the mean. Significant differences in mean dry weight biomass are indicated by differences in letters (*p* < 0.05).

Both the shoot [*F*_(__1,__12__)_ = 30.50, *p* = 0.001] and root spring wheat biomass [*F*_(__1,__12__)_ = 62.64, *p* = 0.001] were significantly impacted by the main effect of soil water availability, whilst an independent effect of P fertilization was not observed (Figure 2). For the roots, significantly lower biomass was observed for spring wheat grown under the higher water (80%) compared to the moderate soil water treatment (60%), regardless of inorganic P fertilization (Figure 2B).

As for roots, regardless of P fertilization, shoot biomass was significantly reduced when grown under the higher soil water treatments (80%) compared to the moderate treatment (60%). An additive reduction in shoot biomass was due to the significant interaction between inorganic P fertilization rate and soil water availability [*F*_(__1,__12__)_ = 7.29, *p* = 0.027] (Figure 2A). This meant that while wheat plants grown in the fertilized soil under the higher soil water treatment (P30-80%) had the lowest shoot biomass (Figure 2A), shoot biomass from unfertilized soil exposed to same water regime (P0-80%) was significantly lower than fertilized soil maintained at the moderate water content (P30-60%) (Figure 2A).

### Soil Bacterial Community Responses at Harvest

#### A Generally Consistent Soil Bacterial Microbiome

No main effect of positioning along the developing root system of spring wheat was detected for bacterial community structure (D1 vs. D5) (Supplementary Table 1). Subsequent analysis of bacterial beta diversity was performed on the sub-section D5 only. Within this sub-section, further comparison between the bulk and rhizosphere soil found the main effect of soil region did not significantly alter bacterial communities (Supplementary Table 2). Data used for further analysis of bacterial beta diversity was therefore from D5 rhizosphere samples and was considered to be representative of bacterial communities within planted soil.

#### A Consistent Interaction Between Soil Water Availability and Plant for Bacterial Beta Diversity

Across the experiment, soil water availability, inorganic P fertilization rate and plant presence had consistent and significant independent main effects on the bacterial community structure at multiple taxonomic levels (Table 2). Analysis of the 2nd order interactive terms revealed an additionally consistent and significant interaction between soil water availability and plant presence (Table 2). The 3rd order interaction was not found to be significant (Table 2).

**TABLE 2:** PERMANOVA results testing for differences between experimental treatments within the D5 sub-section of mesocosms.

	**Phylum**		**Class**		**Family**	
	**Pseudo-F**	***p* (perm)**	**Pseudo-F**	***p* (perm)**	**Pseudo-F**	***p* (perm)**
Phosphorus (P)	2.855	0.037	3.080	0.013	3.623	0.001
Water (W)	6.767	0.003	5.420	0.002	8.805	0.001
Plant (Pl)	4.160	0.015	5.028	0.002	8.841	0.001

P × W	0.782	ns	0.823	ns	0.569	ns
P × Pl	0.702	ns	0.617	ns	1.062	ns
**W** × **Pl**	**4.891**	**0.011**	**5.500**	**0.002**	**7.410**	**0.001**

P × Pl × W	1.226	ns	1.471	ns	1.625	ns

*Soil was sampled from unplanted and planted mesocosms containing field soil that was unfertilized (P0) or had received long-term additions of inorganic phosphorus (P) at 30 kg ha^–1^ y^–1^ (P30) for 44 years. The soil water holding capacity was then adjusted to 60% or 80% and maintained within a growth chamber for 4 months. For planted mesocosms, an individual pre-germinated seed of spring wheat (*T. aestivum*) was sown. Significant differences are highlighted in bold (*p* < 0.05).*

The significant 2nd order interaction between plant presence and soil water was driven by plant presence. The interaction was driven by plant presence, where bacterial community structure differed significantly between planted and unplanted soil only when soil water conditions were moderate (60%), irrespective of long-term inorganic P fertilization (Supplementary Table 3). Additionally, the water regime only significantly impacted bacterial beta diversity when soil was planted. This effect was again regardless of inorganic P fertilization rate (Supplementary Table 4).

#### Individual Responses of Bacterial Taxa

The top three dominant phyla across all treatments were the Firmicutes (35.3%), Acidiobacteria (16.9%), and Proteobacteria (13.3%) (Supplementary Table 5). On average 104 (±18) bacterial families were identified across all experimental treatments ranging from 85 in P0-60% unplanted soil to 143 in P0-60% planted rhizosphere soil.

**FIGURE 3:**
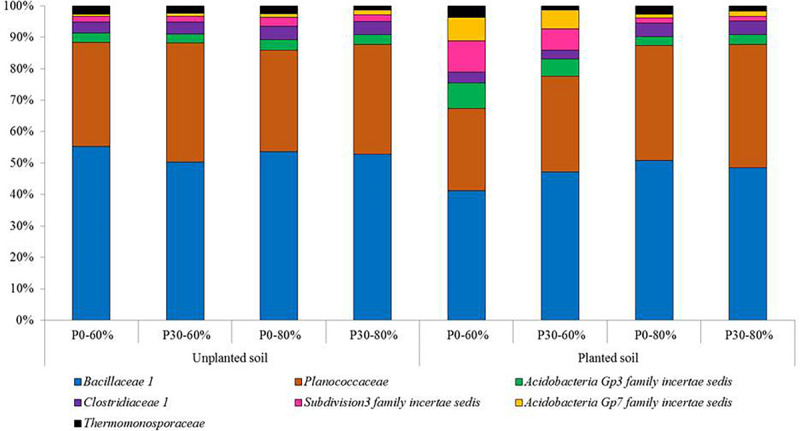
The mean relative abundances of bacterial families significantly responding to experimental treatments from the top 25 most relatively abundant. Experimental treatments are unplanted and planted soil sampled from mesocosms using field soil that was unfertilized (P0) (*n* = 3) or had received long-term additions of inorganic phosphorus (P) at 30 kg ha^–1^ y^–1^ (P30) (*n* = 3) (44 years). The soil water holding capacity was then adjusted to 60% or 80% and was maintained within a growth chamber for four months. For planted mesocosms, an individual pre-germinated seed of spring wheat (*T. aestivum*) was sown and the experiment ran for 4 months within a growth chamber. Samples were sent for targeted amplicon sequencing of the bacterial 16S *rRNA* gene.

Based on the relative abundance data, the top 25 most relatively abundant bacterial families detected across the experiment are presented in Supplementary Table S6. Within this top 25, the individual responses of six bacterial families were found to significantly respond to experimental treatments. These families were, *Bacillaceae 1*, *Clostridiaceae 1*, *Subdivision3 family incertae sedis, Gp7 family incertae sedis, Thermomonosporaceae*, *and Planococcaceae* (Figure 3).

#### Main Effects

For *Subdivision3 family incertae sedis*, the main effects of inorganic P fertilization, soil water availability and plant presence all had significant main effects on the relative abundance of this bacterial family (Table 3). For *Gp7 family incertae sedis*, the relative abundance of this family significantly responded to the two main effects of soil water availability and plant presence (Table 3). Different main effects were significant for *Bacillaceae 1* and *Thermomonosporaceae*, with the plant being significant for *Bacillaceae 1* and inorganic P fertilization significant for *Thermomonosporaceae* (Table 3). *Clostridiaceae 1* and *Planococcaceae* were not significantly influenced by any of the three experimental treatments as main effects.

**TABLE 3:** Test statistics and *p* value summary table for bacterial families within the top 25 most relatively abundant that were significantly affected by main and interactive terms across the experiments.

	**Bacterial families significantly affected by experimental treatments from top 25 most relatively abundant**											
	***Bacillaceae 1***		***Clostridiaceae 1***		***Gp7 family incertae sedis***		***Subdivision3 family incertae sedis***		***Thermomonosporaceae***		***Planococcaceae***	
**Treatment**	***F*_(__1,__24__)_**	***p***	***F*_(__1,__24__)_**	***p***	***F*_(__1,__24__)_**	***p***	***F*_(__1,__24__)_**	***p***	***F*_(__1,__24__)_**	***p***	***F*_(__1,__24__)_**	***p***
P	2.284	ns	1.470	ns	0.079	ns	**3.852**	**0.007**	**28.272**	**0.001**	0.516	ns
W	1.003	ns	2.618	ns	**24.704**	**0.001**	**11.310**	**0.004**	0.206	ns	2.322	ns
Pl	**5.958**	**0.022**	0.893	ns	**57.355**	**0.001**	**6.432**	**0.022**	1.518	ns	1.562	ns

P × W	0.229	ns	0.203	ns	0.468	ns	0.141	ns	0.341	ns	0.414	ns
P × Pl	0.957	ns	0.088	ns	0.826	ns	0.054	ns	0.024	ns	0.313	ns
W × Pl	**5.089**	**0.038**	**6.050**	**0.020**	**38.218**	**0.001**	**16.465**	**0.001**	**8.389**	**0.011**	**10.716**	**0.005**

P × W × Pl	0.574	ns	0.103	ns	1.284	ns	1.097	ns	0.420	ns	0.117	ns

*Relative abundances are from soil receiving long-term differences in inorganic phosphorus (P) fertilization (0 or 30 kg ha^–1^ y^–1^), which were subjected to differential changes in soil water holding capacity (W) (60% or 80% soil WHC). Within these treatments mesocosms were planted with an individual pre-germinated seed of spring wheat (*T. aestivum*), or unplanted. Mesocosms were maintained at these conditions for 4 months within a growth chamber. Significant differences are highlighted in bold (*p* < 0.05).*

#### Interactive Effects

Despite an absence of significant main effects for *Clostridiaceae 1* and *Planococcaceae*, all six bacterial families, *(Bacillaceae 1, Clostridiaceae 1*, *Subdivision3 family incertae sedis, Gp7 family incertae sedis, Thermomonosporaceae*, and *Planococcaceae)* were all significantly affected by a consistent 2nd order interaction between soil water availability and plant presence (Table 3).

For the *Bacillaceae 1, Planococcaceae* and *Clostridiaceae 1* these results manifested themselves by a reduced relative abundance in planted soil maintained at the moderate soil water content (60%) compared to equivalent planted soil exposed to the higher soil water content (80%), regardless of inorganic P fertilization rate. In addition, these three families reduced in relative abundance significantly compared to most unplanted treatments (Supplementary Table 6).

For *Subdivision3 family incertae sedis* and *Gp7 family incertae sedis*, the opposite response was generally observed, with their relative abundances increasing in the planted soil maintained at the moderate soil water content (60%) compared to other treatments including the unplanted treatments (Supplementary Table 6).

For the *Thermomonosporaceae* family, whilst not consistently significant across all P30 treatments, a significant reduction in this bacterial family was observed within some fertilized (P30) soil when compared to unfertilized soils (Supplementary Table 6).

### Soil Enzymatic Activity at Harvest

**FIGURE 4:**
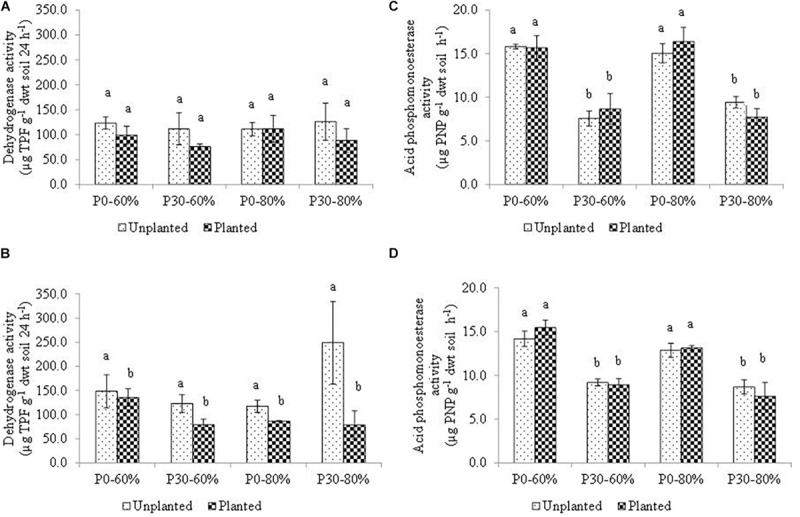
Mean dehydrogenase activity measured in bulk soil within the **(A)** D1 and **(B)** D5 and mean acid phosphomonoesterase measured in bulk soil within the **(C)** D1 and **(D)** D5 sub-sections of sampled mesocosm. Activity was measured after four months incubation in a growth chamber. Soil used in the experiment originated from an established inorganic phosphorus (P) fertilization field trial (44 years) which had either remained unfertilized (P0) or had received 30 kg ha^–1^ y^–1^ (P30) of inorganic P. Soil water holding capacity was altered to 60% and 80% and was maintained within a growth chamber for four months. Error bars represent standard error of the mean (*n* = 3). Significant differences are indicated by letters (*p* < 0.05).

The activity measurements of D1 and D5 bulk soil at the end of the experiment for dehydrogenase and acid phosphomonoesterase activity are presented in Figure 4.

#### Dehydrogenase Activity Along the Soil Profile

Within the top sub-section of the mesocosms, (D1), mean soil dehydrogenase activity did not differ significantly across the experimental treatments as a result of main treatment effects, or due to any of the 2nd, or even the 3rd order interaction (Table 4 and Figure 4A).

**TABLE 4:** Test statistics and *p* value summary table for dehydrogenase and acid phosphomonoesterase.

	**Dehydrogenase activity**				**Acid phosphomonoesterase activity**			
	**D1**		**D5**		**D1**		**D5**	
**Treatment**	***F*_(__1,__24__)_**	***p***	***F*_(__1,__24__)_**	***p***	***F*_(__1,__24__)_**	***p***	***F*_(__1,__24__)_**	***p***
P	0.029	ns	0.085	ns	**110.740**	**0.001**	**68.190**	**0.001**
W	0.214	ns	0.007	ns	0.374	ns	1.913	ns
Pl	3.847	ns	**5.958**	**0.022**	0.035	ns	0.221	ns
P × W	0.310	ns	2.582	ns	0.015	ns	1.407	ns
P × Pl	1.277	ns	3.446	ns	0.267	ns	2.090	ns
W × Pl	0.068	ns	1.252	ns	0.611	ns	0.021	ns
P × W × Pl	1.034	ns	0.170	ns	1.885	ns	0.122	ns

*Measurements are from bulk soil receiving long-term differences in inorganic phosphorus (P) fertilization (0 or 30 kg ha^–1^ y^–1^), which were further subjected to differential changes in soil water (W) availability (60% or 80% soil WHC). Within these treatments mesocosms were both planted and unplanted. Mesocosms were then maintained at these conditions for 4 months within a growth chamber. Significant differences are highlighted in bold (*p* < 0.05).*

At the lower mesocosm sub-section (D5), however, dehydrogenase activity differed significantly due to the main effect of the plant (Table 4), with activity of the unplanted mesocosms showing heightened activity (Figure 4B).

#### Acid Phosphomonoesterase Activity Along the Soil Profile

For acid phosphomonoesterase activity, a significant independent effect of inorganic P fertilization rate was detected, regardless of position descending the soil profile within the mesocosms (D1 and D5) (Figures 4C,D). The result mirrors the baseline field observation prior to experimental manipulation (Table 1). As a result of this sole main effect, a significantly lower acid phosphomonoesterase activity was determined for all fertilized (P30) treatments, compared to all unfertilized (P0) treatment regardless of water regime and plant presence (Figures 4C,D).

## Discussion

The mesocosm study provides insight into complex above and belowground plant-soil-microbial interactions to increased soil water availability and inorganic P fertilization rate. Whilst studies have attempted to model wheat productivity to a range of climate change scenarios (Southworth et al., 2002; Tao and Zhang, 2013; Mohan, 2014; Váry et al., 2015), study of the spring wheat-soil microbiome in this context is unexplored.

Soil used within the study was sampled from an established (44 year), inorganic P fertilization field trial, where differences in total and available inorganic P, soil microbial biomass P and bacterial community structure have been documented (Griffiths et al., 2012; Chen et al., 2014; Randall et al., 2019). The aim of this study was to determine if this effect persisted when combined with a short-term change in soil water availability, whilst exploring the role of the spring wheat rhizosphere as a mediator. Whilst large scale field studies are desirable, parameters such as soil water availability are difficult to control *in situ*. Mesocosm studies therefore allow such variables to be controlled (Williams and Rice, 2007). Albeit not representative of realistic field scenarios, this study lays foundations for future research, in an area currently understudied.

Soil by its very nature is heterogeneous, as such, equal soil WHC and moisture content between our field plots was unlikely. To control this aspect of the experiment, the soil WHC of each field plot was adjusted to two equal levels (60% and 80%), using an experimental approach typical of similar studies (Barratt et al., 2003; Uhlírová et al., 2005; Chen et al., 2007; Dijkstra and Cheng, 2007). Selection of the moderate soil water treatment (60% WHC) simulated “optimal” moisture content for aerobic microbial processes (Papendick and Campbell, 1981). The 80% soil WHC was selected to represent heightened periods of rainfall, as opposed to flooding events (100% WHC) (Barratt et al., 2003).

### Baseline Soil Enzymatic Activity and Soil Characteristics

Despite a lower soil pH in the P0 soils, the average pH of both unfertilized (5.33) and fertilized (P30) (5.64) soils was acidic. This is important, as soil pH is an important driver of inorganic P availability within soils (Alt et al., 2011; Brod et al., 2015) and can be a significant driver of changes in soil microbial communities (Griffiths et al., 2011; Zhalnina et al., 2014).

Measures of soil enzymatic activity can serve as bioindicators of changes within the soil (Henry, 2012; Stone et al., 2016). The two enzymes assayed in the current study provide indication of the P mineralizing (acid phosphomonoesterase) and aerobically active (dehydrogenase) potential of soil (Nannipieri et al., 2011). The significant difference in baseline activity of acid phosphomonoesterase between unfertilized (P0) and P30 fertilized field plots supports the use of this enzymes as an indicator of soil P status. Typically activity increases when available inorganic P concentrations are low (Olander and Vitousek, 2000; Shi et al., 2013; Turner and Joseph Wright, 2014), which is supported by the low concentrations detected at the unfertilized (P0) plots in the current study. The similarity in baseline measurements of dehydrogenase activity between the unfertilized and fertilized (P30) field plots suggests soil aerobic activity has not impacted by long-term differences in soil P status due to P fertilization. Whilst an insensitivity of this enzyme is not uncommon (Chu et al., 2007; Shi et al., 2013), opposing responses have been noted with P fertilization (Beauregard et al., 2010; Luo et al., 2015).

### Bulk Soil Enzymatic Activity at Experimental Harvest

Elevated soil water availability has the ability to impact a large portion of the soil microbial community. Waterlogging events have been shown to decrease redox potential, O_2_ diffusion (Sharma and Swarup, 1988; Yaduvanshi et al., 2012) and concentration to 2% (Belford et al., 1980). As a result, changes to soil processes such as enzyme activities are likely. In the mesocosm experiment, however, the unaltered activity of acid phosphomonoesterase measurements, suggests an insensitivity to elevated water availability compared to long-term differences in soil P status.

As a measure of the aerobic potential of soil, it may be expected for dehydrogenase activity to be negatively impacted by increased soil water availability. Here we failed to observe this. Instead a significant reduction in dehydrogenase activity was measured at the root tip for all planted soil. Whilst difficult to untangle, the rhizosphere can significantly facilitate changes in the soil environment (Luster et al., 2009; Neumann et al., 2009; Philippot et al., 2013), perhaps relevant for dehydrogenase, which is not solely microbial, as is acid phosphomonoesterase. Future work should adopt a molecular functional approach, targeting genes associated with microbial P cycling (Fraser et al., 2014; Ragot et al., 2016) and anaerobic conditions such as methanogenesis (Angel et al., 2011). This will provide a refined measure of the microbial contribution to important soil processes as opposed to broader enzyme responses.

### Response of Spring Wheat Biomass at Experimental Harvest

Typically, metrics used to determine wheat quality and grain yield (grain weight and kernels per ear head) develop during the post vegetative growth stage (Hütsch et al., 2019). Nonetheless, the end of the vegetative growth period can provide significant indication of downstream wheat development (Santos and Diola, 2015). It is therefore important to understand how the vegetative growth of spring wheat can be impacted as it may be useful for early stage wheat management.

Known to be sensitive to changes in climate (Dubey et al., 2019), both waterlogging (Araki et al., 2012; Yaduvanshi et al., 2012) and short-term flooding (Sharma and Swarup, 1988) can impact wheat development. This result was mirrored in the current study at the early growth stage. As a globally significant crop, wheat is used within 21% of food sources (Wu et al., 2004; Ortiz et al., 2008), with recent figures of European production reaching 142.7 million tonnes (Eurostat, 2019). Importantly the interaction between P fertilization and soil water content in the current study, may indicate a negative impact on yield as future precipitation increases across Atlantic Europe (IPCC, 2014). Naturally, this requires testing at a larger scale and over longer periods, but we may see costs for the agri-sector, particularly for soils fertilized by inorganic P, such as cultivated grasslands.

### Response of Bacterial Communities at Experimental Harvest

Plants significantly contribute and mediate C translocation belowground, via the roots and into the rhizosphere (Lakshmanan et al., 2014). This introduction can influence soil heterotrophic respiration, microbial community composition and activity (Hinsinger et al., 2009; Ai et al., 2013; Skwarylo-Bednarz and Krzepilko, 2013; Nie et al., 2014). By sampling at two proximities away from the root system of spring wheat, the ability of the rhizosphere to shape bacterial communities was assessed. Studies comparing bulk and rhizosphere microbial community assemblages from the same samples often observe differences in structure (Ai et al., 2013; Bakker et al., 2015; Praeg et al., 2019). In the current study, however, this was not so. As demonstrated by the effect of cabbage age in shaping bacterial beta diversity (O’Brien et al., 2018), the experimental and plant establishment time may have influenced this. The need for future studies to run for longer, capturing the entire crop growing season is additionally cemented by this.

Despite no distinction between bulk and rhizosphere bacterial community structure during the 4 month experiment, planted and unplanted soil showed clear distinctions. The significant ability of spring wheat to influence this microbial group is clear, and it is only through the plant that soil water content becomes significant. This result demonstrates the complexity of plant-soil-microbial interactions which will require consideration in future crop production (de Vries and Wallenstein, 2017). It also suggests a heightened sensitivity of wheat associated bacterial communities to soil water content, compared to those within unplanted soil. Under drought experiments, it appears bacteria are sensitive to water fluctuations, more so than fungal communities (Borowik and Wyszkowska, 2016), likely driven by changes in root exudation (de Vries et al., 2018; Gargallo-Garriga et al., 2018; Naylor and Coleman-Derr, 2018). Exploration of both root exudation and the wider soil microbiome within the context of our research is therefore welcomed. By demonstrating a strong water-plant interaction within the current study, we have also shown how elevated soil water availability can override the effect of long-term differences in inorganic P fertilization on soil bacterial community structure (Griffiths et al., 2012; Chen et al., 2014; Randall et al., 2019). The use of key bacterial taxa found within unfertilized (P0) soils with potential roles as sustainable modes of P mobilization (Owen et al., 2015; Stamenković et al., 2018) may therefore be impacted under wetter conditions.

### Response of Individual Bacterial Taxa

Collectively, the individual responses of the bacterial families support the significant influence of the spring wheat rhizosphere in shaping the wider bacterial community. The individual responses also support the observation that the plant effect is significantly mediated by the soil water content. These significant responses of individual bacterial taxa may therefore be indicative of changes to communities within future temperate cultivated grassland soils.

Whilst *Bacillaceae and Clostridiaceae* are commonly found in soil (Mandic-Mulec et al., 2015), the comparatively lower relative abundances of these families and *Planococcaceae* in planted soil, at moderate (60%) soil water regimes suggests a potential suppression due to this treatment. Members within the *Bacillaceae* family are mostly aerobic or facultative anaerobic chemoorganotrophs, phylogenetically similar to the *Bacillaceae* family (Shivaji et al., 2014). The *Clostridiaceae*, however, are strict anaerobes (Mandic-Mulec et al., 2015). Organisms belonging to both *Bacillaceae and Clostridiaceae* families can exhibit increased tolerance to environmental stressors. This is due to their endospore forming capabilities (Nessner Kavamura et al., 2013), with *Clostridiaceae* important within the pre-treatment consortia of anaerobic digesters to enhance CH_4_ production from plant waste (Suksong et al., 2019). Our response within temperate grassland soil for this family is complemented by significantly greater relative abundance within both anoxic regions of rice paddy fields (Lüdemann et al., 2000), but also upon soil re-wetting events (Liesack et al., 2000; Noll et al., 2005; Reim et al., 2017).

Conversely, the two bacterial families that significantly increased within the same treatment (60% planted soil) belong to the phylum Acidobacteria. Acidobacteria are commonly profiled in grassland soil (Quaiser et al., 2003; da Rocha et al., 2013) and whilst relatively little is known about this diverse phylum, Acidobacteria are detected across a range of habitats (Quaiser et al., 2003; Naether et al., 2012) and are highly abundant (Janssen, 2006), particularly in acidic soils (Jones et al., 2009; Griffiths et al., 2011; Dedysh et al., 2017) as used in the current study. Our results reflect this phylum’s ability to dominate environments with reduced substrate inputs such as the P0 soil (Naether et al., 2012), but they do not fully support it, due to an insensitivity to P fertilization. Previous studies have noted both positive and negative responses by members of the Acidobacteria to soil moisture (Naether et al., 2012). The negative response to heightened water availability observed in planted mesocosms suggests a potentially important sensitivity.

Finally, varied response in the relative abundance of the family *Thermomonosporaceae* across treatments suggests a complex interaction with plant and soil water availability, influenced by P fertilization or other soil parameters. Unfortunately, limited knowledge surrounds *Thermomonosporaceae* in terms of responses to environmental perturbations, however, the phylum Actinobacteria, to which it belongs is associated with increased solubilization of inorganic P (Mander et al., 2012). The general decreased relative abundance of *Thermomonosporaceae* to high P fertilization may support this, however, it is not a significant observation.

## Conclusion

This study has attempted to address a knowledge gap in soil-microbial-climate change research that is relevant for Atlantic Europe. Clearly much work is still needed in this area, but, the fact a relatively short-term increase in soil water availability and plant significantly overrode effects of long-term inorganic P fertilization is interesting for the future health of temperate cultivated grassland soils.

## Data Availability Statement

The datasets generated for this study can be found in the NCBI SRA repository, with accession number PRJNA610548.

## Author Contributions

KR designed the experiment. Design, sampling logistics and sampling were discussed with ED, NC, BG, FB, and RC as was analyses and manuscript preparation. KR set-up and sampled the experiment. KR also conducted the lab work, with SS helping with NGS library preparation and bioinformatics. KR conducted main portion of the bioinformatics for NGS data and all statistical analyses.

## Conflict of Interest

The authors declare that the research was conducted in the absence of any commercial or financial relationships that could be construed as a potential conflict of interest.
