# Supplementary material for: An Assessment of Climate Induced Increase in Soil Water Availability for Soil Bacterial Communities Exposed to Long-Term Differential Phosphorus Fertilization
Source: Front Microbiol. 2020 May 15;11:682. doi: 10.3389/fmicb.2020.00682 (PMC7242630; doi:10.3389/fmicb.2020.00682)
Supplement: Supplementary file 1 [file Data_Sheet_1.pdf]

Supplementary Material

In order to alter the soil water holding capacity (WHC), the gravimetric water content and the soil field capacity needed to be determined. These percentages used in the experiment were chosen based on earlier studies investigating ecological impacts of elevated soil WHC (Marković et al., 2015; Chen et al., 2007; Dijkstra & Cheng, 2007).

Calculating gravimetric soil water content

A tin foil weight boat was weighed, and mass recorded (w1). 5 g of the sieved soil was added to a weigh boat and weight recorded. The mass of the weigh boat (w1) + was then subtracted from the wet soil (g) + weigh boat to derive the mass of fresh weight soil (w2). Samples were then placed in an oven at 105 ^{o}C for 24 h, after which time, the mass (g) of the dry soil + the weigh boat was recorded. To determine soil dry weight (g), w1 was subtracted from this final mass (w3). The gravimetric water content of the soil was then calculated as follows and expressed as a percentage:

$$\text{Gravimetric moisture content (mc \%)} = \left(\frac{\text{w2-w3}}{\text{w2}}\right) * 100$$

Soil field capacity

Soil field capacity is defined as the gravimetric water content of sieved soil that remains after soils has been saturated under gravity (Robertson et al., 1999). A plug of glass wool was placed into a Haines funnel system and moistened with deionised water and the silicon tubing clamped shut. 50 g of the fresh sieved soil was then placed in the funnel and 100 ml deionised water added to the soil. Tin foil was then placed over the top of the funnel to prevent evaporation. After 24 h at room temperature the clamp was opened. The water was allowed drain into a measuring cylinder for 30 min and the volume collected was measured. Using the pre-determined soil gravimetric moisture content data, the percentage of the field capacity the soil was determined (Jenkinson, D. and Powlson, S., 1976)

$$\frac{(\text{Extra } H_2O \text{ per *100g soil + mc \%})}{\text{100g soil + extra } H_2O}$$

(This accounts for the 100 g of soil you are working with, plus the extra H_2O retained).

Adjusting soil WHC

To adjust the soil WHC, the percentage of field capacity that the fresh soil is at in the current sieved state must be determined by the following equation:

$$\left(\frac{\text{mc \%}}{\text{Soil WHC}}\right) *100$$

Then calculate:

(A) New gravimetric moisture content needed to reach the target soil WHC =

$$\left(\frac{\text{(Target soil WHC (\%) * Current mc \%)}}{\text{Current soil whc of sieved soil}}\right) * 100$$

(B) New amount of H_2O required to achieve new mc % =

$$\frac{\text{(New gravimetric moisture content for target soil WHC (\%) * dry weight soil)}}{\text{(New gravimetric moisture content for target soil WHC (\%) for target soil whc (\%) -1)}}$$

(The -1 converts to a positive number).

(C) Calculate how much H_2O needs to be added (+) or lost (-) to reach desired soil WHC

H_2O to reach new gravimetric moisture content for target soil WHC - H_2O already in the soil

If the current gravimetric soil water content is too high, soil must be laid out in a tray and allowed to air dry checking the moisture content every 24 h.

If water is required to be added to reach the desired soil WHC, soil was added to trunking of known mass and weighed. The soil was sprayed with distilled water with the amount required to reach the desired soil WHC and then re-weighed. The mass of the trunking and soil was then maintained throughout the experiment. To maintain the water content, the front of the trunking was removed, and distilled water was slowly added evenly along the profile.

Once set-up was complete, the weight of each mesocosm was recorded. For planted mesocosms, one seed of spring wheat *(T. aestivum)* Trappe variety (Goldcrop Ltd, Co. Cork, Ireland) was sown within the soil. Distilled water was used to water mesocosms throughout the experiment by removing the front. The volumes of water used to maintain water levels throughout the experiment were determined by subtracting the initial mean weight of the unplanted mesocosms from each mesocosm per treatment.

(a) (b)

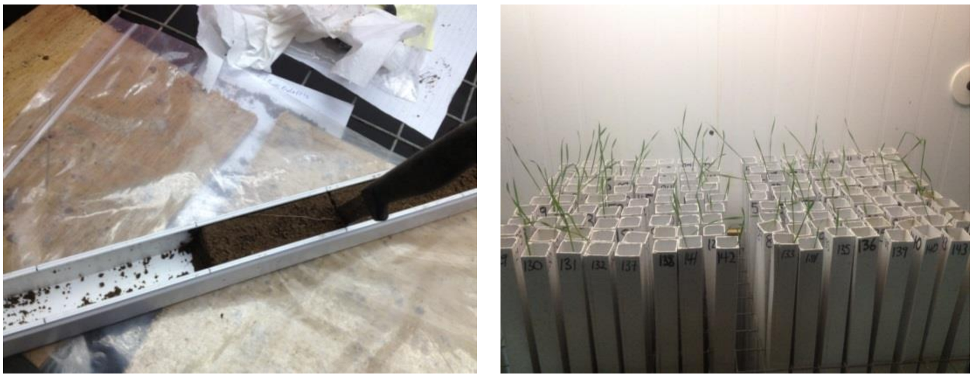

S Figure 1 (a) An individual mesocosm demonstrating how the front cover was removed to add soil, water and for sampling and (b) mesocosms collectively positioned within the growth chamber.

S Table 1 Pair-wise comparison testing for differences in bacterial community structure between the D1 and D5 mesocosm sections at the family level. Samples were taken from mesocosms filled with soil from a long-term inorganic phosphorus (P) fertilisation field trial. Soil had either remained unfertilised (P0), or fertilised with inorganic P (P30) 30 kg ha^{-1} y^{-1}. The soil water holding capacity was adjusted to 60% or 80% and maintained for four months within a growth chamber. Mesocosms were either sown with a single seed of spring wheat (*T. aestivum*, or remained unplanted. The *p* values were generated from the Monte Carol tests ($p<0.05$).

**Mesocosm sub-section**	**Treatment**	**t**	***p* (MC)**
Unplanted soil D1 vs. D5	60% - Unplanted P0	1.440	ns
	60% – Unplanted P30	1.375	ns
	80% - Unplanted P0	1.123	ns
	80% - Unplanted P30	1.009	ns
Rhizosphere soil D1 vs. D5	60% - Planted P0	1.447	ns
	60% – Planted P30	1.345	ns
	80% - Planted P30	1.459	ns
	80% - Planted P0 x P30	2.066	ns

S Table 2 Pair-wise comparisons testing for differences in planted rhizosphere and bulk soil bacterial community structure sampled from the D5 mesocosm section identified at phylum, class and genus level. Samples were taken from mesocosms filled with field soil that remained unfertilised for 44 years (P0), or had received fertilisation (30 kg ha^{-1} y^{-1}) (P30). The soil water holding capacity was adjusted to 60% or 80% and maintained for four months within a growth chamber. Mesocosms were either sown with a single seed of spring wheat (*T. aestivum*, or remained unplanted (p<0.05).

	**Phylum**		**Class**		**Family**	
**Planted soil (D5)**	t	P(MC)	t	P(MC)	t	P(MC)
P0–60% Bulk x Rhizosphere soil	1.755	ns	1.239	ns	0.969	ns
P0-80% Bulk x Rhizosphere soil	0.653	ns	0.873	ns	0.522	ns
P30-60% Bulk x Rhizosphere soil	0.970	ns	1.189	ns	1.163	ns
P30–80% Bulk x Rhizosphere soil	0.900	ns	1.036	ns	0.749	ns

S Table 3 Pair-wise comparison testing for differences in the community structure of bacterial families between planted and unplanted soil sampled from the D5 mesocosm section, taxonomically classified at phylum, class and family levels. Samples were taken from mesocosms filled with field soil that had received no inorganic phosphorus fertilisation (P0), or 30 kg ha^{-1} y^{-1} (P30). The soil water holding capacity was adjusted to 60% or 80% and maintained for four months within a growth chamber. Mesocosms were either sown with a single seed of spring wheat (*T. aestivum*, or remained unplanted. Significant differences are highlighted in bold (p<0.05).

**Plant**	**t**	***p* (MC)**
P0-60% Unplanted x Planted	**3.383**	**0.001**
P0-80% Unplanted x Planted	0.596	ns
P30-60% Unplanted x Planted	**2.999**	**0.011**
P30-80% Unplanted x Planted	1.292	ns

S Table 4 Pair-wise comparison testing for differences in bacterial community structure between water treatments sampled from the D5 mesocosm section, taxonomically classified at phylum, class and family levels. Samples were taken from mesocosms filled with field soil that had received no inorganic phosphorus fertilisation (P0), or 30 kg ha^{-1} y^{-1} (P30). The soil water holding capacity was then adjusted to 60% or 80% and maintained for four months within a growth chamber. The planted samples had one seed of spring wheat (*T. aestivum*) sown into the soil. Significant differences are highlighted in bold (p<0.05).

**Water**	**t**	***p* (MC**
P0 Unplanted 60% x 80%	0.835	ns
P0 Planted 60% x 80%	**3.457**	**0.005**
P30 Unplanted 60% x 80%	1.081	ns
P30 Planted 60% x 80%	**3.297**	**0.010**

S Table 5 Bacterial phyla contributing to >1% of the total relative abundance. Samples were taken from mesocosms filled with field soil that had received no inorganic phosphorus (P) fertilisation (P0), or 30 kg ha^{-1} y^{-1} (P30). The soil water holding capacity was adjusted to 60% or 80% and maintained for four months within a growth chamber. The planted samples had one seed of spring wheat (*T. aestivum*) sown into the soil (p<0.05). Data presented are mean relative abundances (n=3).

	Unplanted soil				Planted soil				Relative abundance (%)	Cumulative relative abundance (%)
	P0-60%	P30-60%	P0-80%	P30-80%	P0-60%	P30-60%	P0-80%	P30-80%		
Firmicutes	47.6 a	39.0 abc	41.9 abc	45.0 ab	22.0 c	21.6 bc	42.3 ab	38.3 abc	35.3	35.3
Acidobacteria	11.5 c	15.5 bc	14.7 c	15.2 c	21.1 ab	23.2 a	13.3 c	15.0 c	16.9	52.1
Proteobacteria	11.2 c	10.1 c	10.2 c	9.0 c	18.4 a	16.0 ab	11.9 c	13.0 bc	13.3	65.4
Unclassified	8.4	12.3	11.0	11.2	12.7	14.4	9.4	11.8	11.6	77.0
Verrucomicrobia	9.4 abc	7.9 abc	8.9 abc	6.4 c	13.0 ab	13.3 a	6.9 abc	7.1 bc	9.3	86.3
Actinobacteria	8.2 ab	12.2 a	9.1 ab	10.9 ab	6.6 b	5.5 b	10.0 ab	12.1 a	9.1	95.4
Planctomycetes	2.5 a	2.1 b	2.8 a	1.3 b	2.7 a	2.1 b	2.1 a	1.7 b	2.3	97.7
Bacteroidetes	0.5 b	0.2 b	0.5 b	0.3 b	2.1 a	1.6 b	3.2 a	0.3 b	1.1	98.8
Chloroflexi	0.2 a	0.3 a	0.3 a	0.4 a	0.4 a	0.7 a	0.5 a	0.3 a	0.2	99.2
Gemmatimon...	0.2 a	0.2 a	0.2 a	0.1 a	0.3 a	0.2 a	0.2 a	0.2 a	0.2	99.4
Nitrospira	0.1 a	0.2 b	0.1 a	0.1 b	0.2 a	0.3 a	0.1 a	0.2 a	0.2	99.6
WS3	0.1 ab	0.1 b	0.1 ab	0.1 b	0.3 a	0.3 ab	0.1 b	0.1 b	0.2	99.7

S Table 6 Relative abundances of bacterial families profiled in planted and unplanted soil sampled from the D5 mesocosm section of mesocosms using field soil that had remained unfertilised (P0) or had received 30 kg ha^{-1} y^{-1} inorganic phosphorus (P) (P30). The soil water holding capacity was adjusted to 60% or 80% and maintained for four months within a growth chamber. The planted samples had one seed of spring wheat (*T. aestivum*) sown into the soil. Data presented are mean relative abundances (n=3). Significant differences are highlighted in bold (p<0.05).

	**Unplanted soil**				**Planted soil**			
	**P0-60%**	**P30-60%**	**P0-80%**	**P30-80%**	**P0-60%**	**P30-60%**	**P0-80%**	**P30-80%**
Unclassified	31.88 (*0.90*)	36.43 (*1.32*)	34.17 (*1.21*)	23.12 (*11.39*)	35.99 (*1.88*)	35.32 (*0.47*)	34.32 (*0.71*)	37.34 (*0.73*)
Bacillaceae_1	**19.62 (*2.14*) [a]**	**13.96 (*2.62*)[a]**	**16.49 (*2.63*)[a]**	**11.96 (*6.43*)[ab]**	**7.70 (*1.37*)[b]**	**9.15 (*1.42*)[ab]**	**16.90(*0.51*)[a]**	**13.28 (*1.76*)[a]**
Planococcaceae	**11.71 (*0.67*) [a]**	**10.49 (*1.78*)[ab]**	**9.89 (*1.22*)[a]**	**7.91 (*4.14*) [ab]**	**4.89 (*0.78*)[b]**	**5.93 (*0.55*)[b]**	**12.17(*0.94*)[a]**	**10.73 (*1.14*)[a]**
Spartobacteria_family_incertae_sedis	8.60 (*1.32*) [a]	7.30 (*1.44*) [a]	7.93 (*0.68*) [a]	3.92 (*4.99*) [a]	10.93(*2.05*)[a]	12.05 (*0.17*) [a]	7.13 (*1.25*) [a]	6.88 (*1.12*) [a]
Acidobacteria_Gp6_family_incertae_sedis	4.18 (*0.72*) [a]	6.05 (*0.62*) [a]	5.28 (*0.78*) [a]	4.21 (*2.20*) [a]	8.43 (*1.01*) [a]	10.34 (*0.68*) [a]	5.14 (*0.45*) [a]	5.80 (*0.20*) [a]
Acidobacteria_Gp16_family_incertae_sedis	4.44 (*0.76*) [a]	6.74 (*0.53*)[a]	6.61 (*0.41*) [a]	4.34 (*2.52*) [a]	5.07 (*0.59*) [a]	4.65 (*0.34*) [a]	5.03 (*0.62*) [a]	5.68 (*0.15*) [a]
Planctomycetaceae	2.48 (*0.59*) [a]	2.07 (*0.50*)[a]	2.78 (*0.97*) [a]	0.91 (*0.45*) [a]	2.95 (*0.23*) [a]	2.21 (*0.29*) [a]	2.12 (*0.08*) [a]	1.57 (*0.28*) [a]
Hyphomicrobiaceae	1.25 (*0.21*) [a]	1.26 (*0.35*)[a]	0.85 (*0.21*) [a]	0.47 (*0.23*) [a]	1.18 (*0.07*) [a]	1.35 (*0.18*) [a]	0.98 (*0.09*) [a]	1.74 (*0.17*) [a]
Acidobacteria_Gp3_family_incertae_sedis	1.05 (*0.13*) [a]	0.81 (*0.10*) [a]	0.99 (*0.16*) [a]	0.70 (*0.39*) [a]	1.49 (*0.18*)[s]	1.05 (*0.29*)[a]	0.96 (*0.19*) [a]	0.84 (*0.06*) [a]
Clostridiaceae_1	**1.25 (*0.48*) [a]**	**1.05 (*0.09*) [a]**	**1.34 (*0.22*) [a]**	**0.93 (*0.49*)[ab]**	**0.66 (*0.10*) [b]**	**0.55 (*0.03*) [b]**	**1.43 (*0.32*) [a]**	**1.16 (*0.18*)[a]**
Acidobacteria_Gp1_family_incertae_sedis	0.64 (*0.14*) [a]	0.39 (*0.05*) [a]	0.55 (*0.06*) [a]	0.10 (*0.10*) [a]	2.09 (*0.19*) [a]	1.11 (*0.18*) [a]	0.69 (*0.07*) [a]	0.36 (*0.05*) [a]
Subdivision3_family_incertae_sedis	**0.67 (*0.13*) [b]**	**0.51 (*0.10*) [b]**	**0.88 (*0.27*) [a]**	**0.51 (*0.26*) [b]**	**1.86 (*0.42*) [a]**	**1.32 (*0.12*) [a]**	**0.52 (*0.08*)[b]**	**0.41 (*0.06*) [b]**
Bradyrhizobiaceae	1.18 (*0.20*) [a]	0.58 (*0.06*) [a]	0.80 (*0.16*) [a]	0.22 (*0.15*) [a]	1.01 (*0.07*) [a]	0.73 (*0.07*) [a]	0.99 (*0.15*) [a]	0.95 (*0.08*) [a]
Paenibacillaceae_2	0.83 (*0.09*) [a]	1.03 (*0.21*) [a]	0.99 (*0.10*) [a]	0.80 (*0.46*) [a]	0.41 (*0.05*) [a]	0.46 (*0.06*) [a]	1.08 (*0.16*) [a]	1.09 (*0.05*) [a]
Peptostreptococcaceae	0.94 (*0.18*) [a]	0.85 (*0.17*) [a]	0.79 (*0.04*) [a]	0.90 (*0.47*) [a]	0.30 (*0.02*) [a]	0.40 (*0.05*) [a]	0.74 (*0.10*) [a]	0.98 (*0.10*) [a]
Acidobacteria_Gp7_family_incertae_sedis	**0.20 (*0.05*) [a]**	**0.28 (*0.11*) [a]**	**0.33 (*0.05*) [a]**	**0.34 (*0.18*) [a]**	**1.38 (*0.14*) [b]**	**1.15 (*0.05*)[b]**	**0.41 (*0.11*) [a]**	**0.46 (*0.07*) [a]**
Thermomonosporaceae	**0.96 (*0.11*) [a]**	**0.64 (*0.12*) [a]**	**0.79 (*0.11*) [a]**	**0.29 (*0.15*)[b]**	**0.69 (*0.07*) [a]**	**0.28 (*0.08*) [b]**	**0.88 (*0.15*) [a]**	**0.47 (*0.04*)[ab]**
Acidobacteria_Gp4_family_incertae_sedis	0.20 (*0.07*) [a]	0.32 (*0.08*) [a]	0.25 (*0.07*) [a]	0.22 (*0.11*) [a]	0.85 (*0.08*) [a]	1.38 (*0.08*) [a]	0.19 (*0.06*) [a]	0.26 (*0.07*) [a]
Paenibacillaceae_1	0.52 (*0.15*) [a]	0.64 (*0.12*) [a]	0.53 (*0.05*) [a]	0.39 (*0.19*) [a]	0.27 (*0.05*) [a]	0.31 (*0.03*) [a]	0.61 (*0.12*) [a]	0.46 (*0.06*) [a]
Micromonosporaceae	0.43 (*0.04*) [a]	0.68 (*0.10*) [a]	0.55 (*0.13*) [a]	0.22 (*0.16*) [a]	0.31 (*0.01*) [a]	0.31 (*0.02*) [a]	0.61 (*0.11*) [a]	0.54 (*0.05*) [a]
Chitinophagaceae	0.31 (*0.06*) [a]	0.08 (*0.01*) [a]	0.29 (*0.12*) [a]	0.06 (*0.03*) [a]	1.31 (*0.23*) [a]	0.92 (*0.24*) [a]	0.12 (*0.03*) [a]	0.12 (*0.07*) [a]
Mycobacteriaceae	0.32 (*0.07*) [a]	0.62 (*0.04*) [a]	0.37 (*0.07*) [a]	0.12 (*0.07*) [a]	0.25 (*0.03*) [a]	0.22 (*0.04*) [a]	0.35 (*0.08*) [a]	0.56 (*0.04*) [a]
Xanthomonadaceae	0.19 (*0.03*) [a]	0.19 (*0.01*) [a]	0.20 (*0.04*) [a]	0.16 (*0.08*) [a]	0.71 (*0.11*) [a]	0.50 (*0.14*) [a]	0.29 (*0.03*) [a]	0.20 (*0.02*) [a]
Acidobacteria_Gp17_family_incertae_sedis	0.11 (*0.03*) [a]	0.19 (*0.13*) [a]	0.17 (*0.04*) [a]	0.18 (*0.18*) [a]	0.44 (*0.09*) [a]	0.70 (*0.18*) [a]	0.20 (*0.05*) [a]	0.29 (*0.11*) [a]

References

Chen, M.-M., Zhu, Y.-G., Su, Y.-H., Chen, B.-D., Fu, B.-J., Marschner, P., 2007. Effects of soil moisture and plant interactions on the soil microbial community structure. Eur. J. Soil Biol. 43, 31–38. doi:http://dx.doi.org/10.1016/j.ejsobi.2006.05.001

Dijkstra, F.A., Cheng, W., 2007. Moisture modulates rhizosphere effects on C decomposition in two different soil types. Soil Biol. Biochem. 39, 2264–2274. doi:http://dx.doi.org/10.1016/j.soilbio.2007.03.026

Jenkinson, D. S., Powlson, D.S., 1976. Effects of biocide treatments on metabolism in soil - V Fumigation with chloroform. Soil Biol Biochem 8, 209–213.

Marković, M., Filipović, V., Legović, T., Josipović, M., Tadić, V., 2015. Evaluation of different soil water potential by field capacity threshold in combination with a triggered irrigation module. Soil Water Res. 10, 164–171. doi:10.17221/189/2014-SWR

Robertson, G. P., Coleman, D. C., Bledsoe, C. S., Sollins, P., 1999. *Standard Soil Methods for Long-Term Ecological Research*. Oxford University Press.
